# Effects of Kiwifruit Consumption on Blood Pressure in Adults: A Systematic Review and Meta‐Analysis

**DOI:** 10.1002/hsr2.73126

**Published:** 2026-08-26

**Authors:** Shirin Ghotboddin Mohammadi, Neda Haghighat, Azadeh Dehghani, Mohammad Mahdi Rafiei, Pedram Pam, Mohammadreza Amirkhan‐Dehkordi, Mohammad Ali Goudarzi, Omid Asbaghi, Moslem Naderian, Ali Hosseini, Mehdi Karimi

**Affiliations:** ^1^ Department of Clinical Nutrition, School of Nutrition and Food Sciences Isfahan University of Medical Sciences Isfahan Iran; ^2^ Laparoscopy Research Center Shiraz University of Medical Sciences Shiraz Iran; ^3^ Micronutrient Research Center, Research Institute for Endocrine Disorders, Research Institute for Endocrine Sciences Shahid Beheshti University of Medical Sciences Tehran Iran; ^4^ School of Kinesiology and Physical Activity Sciences Université de Montréal Montréal Canada; ^5^ Department of Clinical Nutrition Tabriz University of Medical Sciences Tabriz Iran; ^6^ Department of Physical Education and Sport Sciences Islamic Azad University Tehran Iran; ^7^ Faculty of Veterinary Medicine University of Lisbon Lisbon Portugal; ^8^ Cancer Research Center Shahid Beheshti University of Medical Sciences Tehran Iran; ^9^ Department of Pharmacognosy, School of Pharmacy Shiraz University of Medical Sciences Shiraz Iran; ^10^ Medicinal Plants Research Center Yasuj University of Medical Sciences Yasuj Iran; ^11^ Faculty of Medicine Bogomolets National Medical University (NMU) Kyiv Ukraine

**Keywords:** blood pressure, Chinese gooseberry, hypertension, kiwifruit, meta‐analysis, nutrition, systematic review

## Abstract

**Background:**

Hypertension represents a major public health challenge worldwide. Nutritional strategies, particularly increased fruit consumption, are being explored as non‐pharmacological means of regulating blood pressure (BP). Kiwifruit, a fruit high in potassium and antioxidant compounds, has been proposed to exert antihypertensive effects; however, findings from individual studies have been inconclusive.

**Aim:**

This meta‐analysis assesses the impact of kiwifruit consumption on BP in adults.

**Method:**

A comprehensive search of online databases was conducted up to May 2026 to identify relevant randomized controlled trials (RCTs). Pooled effect sizes were calculated as weighted mean differences (WMDs) with 95% confidence intervals (CIs) using a random‐effects model to account for between‐study variability.

**Result:**

The pooled analysis of six RCTs (comprising 10 intervention arms) indicated that the consumption of kiwifruits led to a significant reduction in both systolic BP (WMD: −5.73 mmHg, 95%CI: −9.02 to −2.44, *p* = 0.001) and diastolic BP (WMD: −4.08 mmHg, 95%CI: −6.07 to −2.10, *p* < 0.001). Subgroup analysis showed that kiwifruit consumption significantly reduced BP, particularly among individuals with normal BMI, those receiving higher doses (3/day), and male participants. The effect was more pronounced for those with elevated baseline BP and in shorter trials (≤ 8 weeks). Significant differences were observed between subgroups in terms of dose and BMI.

**Conclusion:**

Kiwifruit consumption may contribute to BP reduction as a dietary intervention, particularly among individuals with elevated blood pressure and normal BMI. However, these findings should be interpreted with caution due to the limited number of available RCTs and require confirmation in larger, well‐designed studies.

**Abbreviations**
BP: blood pressure
DBP: diastolic blood pressure
RCT: Randomized Clinical Trial
SBP: systolic blood pressure

## 1 Introduction

Blood pressure (BP) is a persistent and critical risk factor for cardiovascular disease (CVD), heavily influenced by lifestyle choices. Strategies such as weight loss, increased physical activity, reduced alcohol intake, reduced sodium intake, and increased potassium intake are well‐established recommendations for reducing BP [1, 2]. In addition to pharmacological treatments, dietary supplementation also plays a vital role in BP management [3, 4, 5]. The Dietary Approaches to Stop Hypertension (DASH) studies demonstrated that diets rich in fruits, vegetables, and low‐fat dairy products significantly reduce BP [2]. Substantial evidence supports increasing fruit and vegetable consumption to help control BP [6, 7]. Though the recommended intake often exceeds what is typically consumed in Western diets [8]. Potential mechanisms underlying these effects include increased potassium intake and nitric oxide (NO)‐mediated vasodilation. Moreover, oxidative stress has been proposed as a contributing factor in the development of hypertension [9].

Kiwifruit is well‐regarded for its nutritional value, being a rich source of fiber, potassium, vitamin C, and other antioxidants, including lutein, a potent oxycarotenoid [10, 11, 12]. Research has shown that kiwifruit possesses properties beneficial for heart health [13]. Studies such as the Oslo Antioxidant Study have demonstrated that daily consumption of 3 kiwifruits can reduce BP and increase antioxidant capacity. Furthermore, it has been found to upregulate genes involved in stress defense and DNA repair [14]. Kiwifruit also inhibits angiotensin‐converting enzyme [15] activity. Extensive research over the past decade has highlighted the favorable effects of regular kiwifruit consumption on nutritional well‐being and on digestive, immune, and metabolic health. The health benefits associated with fruit consumption have been extensively recorded through academic research [16, 17, 18]. Kiwifruits are rich in vitamin C and also provide significant levels of dietary fiber, potassium, vitamin E, and folate, in addition to various bioactive components such as antioxidants, phytonutrients, and enzymes. Furthermore, they contain essential nutrients, including fat, carbohydrates (such as sugar and dietary fiber), protein (lutein and zeaxanthin), vitamins A, B, C, E, and K, minerals, flavonoids, polyphenols, inositol, and carotenoids. Due to their high vitamin and antioxidant content, kiwifruits may contribute to improved metabolic health [19, 20].

A recent study on acute dietary intervention with kiwifruit showed that post‐meal blood sugar and insulin levels in healthy Chinese individuals decreased after consuming two kiwifruits 30 min before a typical breakfast [21]. This discovery holds significance as previous research has indicated that elevated fasting insulin levels are associated with an increased risk of metabolic syndrome in Asian populations [22, 23]. Therefore, the potential benefits of consuming two kiwifruits before breakfast on immediate post‐meal health outcomes could indicate a simple and effective strategy if maintained over an extended period. Previous studies on dietary interventions have suggested that a duration of 3‐8 weeks is adequate to observe improvements in blood sugar levels, lipid profiles, and BP [24, 25]. Asian individuals are an appropriate population for studying the metabolic and BP impacts of kiwifruit intervention, given their comparatively lower fruit consumption compared to other demographic groups [26]. Additionally, a Japanese study found an inverse relationship between fruit consumption and the risk of hypertension [27].

Given the inconsistent findings across individual randomized controlled trials (RCTs) and the lack of a meta‐analysis focused specifically on BP outcomes, we conducted this systematic review and meta‐analysis to quantitatively evaluate the effects of kiwifruit consumption on systolic and diastolic BP in adults.

## 2 Methods

### 2.1 Study Design and Protocol

This study was conducted in accordance with the PRISMA (Preferred Reporting Items for Systematic Reviews and Meta‐Analyses) statement [28]. The study was structured according to the PICOS [29], as follows:

− **P** (Population): Adults
− **I** (Intervention): Kiwifruit consumption
− **C** (Comparison): Habitual diet, no supplementation, or control
− **O** (Outcomes): Changes in systolic blood pressure (SBP) and diastolic blood pressure (DBP).
− **S** (Study design): RCT

### 2.2 Search Strategy

A comprehensive literature search was conducted in the electronic databases PubMed, ISI Web of Science, and Scopus to identify relevant RCTs and recent systematic reviews on kiwifruit consumption in adult populations, with coverage through May 2026. The search strategy incorporated a combination of keywords and MeSH terms related to the intervention and study design, including: (kiwi OR kiwifruit OR “Actinidia chinensis” OR “Actinidia deliciosa” OR “Actinidia kolomikta” OR “Chinese gooseberry”) AND (randomized OR random OR randomly OR placebo OR “randomized controlled trial” OR “randomized clinical trial” OR RCT OR “Cross‐Over” OR parallel). No restrictions were applied regarding publication date or language. In addition to the database search, a manual search was performed using Google Scholar, and the reference lists of relevant review articles were screened to identify any additional eligible studies.

### 2.3 Study Selection and Eligibility Criteria

Two independent researchers (S.H. G.H. and N.H.) conducted a comprehensive review of relevant articles, screening titles, abstracts, reference lists, and full texts to identify studies that met the eligibility criteria. The inclusion criteria were as follows [1]: studies involving adult participants aged 18 years or older [2]; interventions in which participants consumed kiwifruits for a minimum of 2 weeks [3]; inclusion of a control group where the only variable differing from the intervention was kiwifruit consumption [4]; reporting of outcomes related to SBP and DBP; and [5] use of a RCT design.

The exclusion criteria were [1]: studies involving participants under 18 years of age [2]; use of kiwifruit in combination with other fruits, supplements, or substances as part of a multicomponent intervention [3]; manipulation of the kiwifruit (e.g., use of extracts or processed forms) [4]; studies lacking a control group or appropriate comparator [5]; non‐trial study designs (e.g., observational studies, case reports); and [6] insufficient or missing data on BP outcomes.

### 2.4 Risk of Bias Assessment

The methodological quality of the included RCTs was assessed using the Cochrane Risk of Bias Tool version 1, as recommended by the Cochrane Handbook for Systematic Reviews of Interventions [30]. Two independent reviewers (O.A. and M.K.) evaluated each study across seven domains: random sequence generation, allocation concealment, blinding of participants and personnel, blinding of outcome assessment, incomplete outcome data, selective reporting, and other potential sources of bias. Each domain was rated as low, unclear, or high risk of bias, and the overall study quality was categorized accordingly as low, moderate, or high risk. Disagreements were resolved through discussion or consultation with a third reviewer to ensure reliability and minimize subjectivity in the assessment.

### 2.5 Data Extraction

Two researchers (P.P. and A.J.) independently performed data extraction from the selected RCTs. The information gathered included the first author's name, year of publication, study location, study design and methodology, participant characteristics (including sex and sample size per group), trial duration, mean age, body mass index (BMI), details of the intervention and its dosage, along with the mean and standard deviation values of SBP and DBP recorded before and after the intervention. All extracted data were organized and consolidated into a single Excel file for further analysis.

### 2.6 Statistical Analysis

In this meta‐analysis, statistical analyses were conducted in Stata (version 14; StataCorp LP) to evaluate the data. Overall effect sizes were determined by extracting weighted mean differences (WMD) and standard deviations (SD) from both the intervention and control groups, and these values were analyzed using a random‐effects model based on the DerSimonian and Laird method [31]. Changes in SBP and DBP from baseline to the end of the trial were calculated between the intervention and control groups. Predefined subgroup analyses were performed according to baseline SBP and DBP levels, trial duration (≥ 8 vs. < 8 weeks), kiwifruit consumption dose, baseline BMI (kg/m^2^), and participant sex (male vs. female). Sensitivity analyses were conducted to assess the robustness of the findings by excluding one study at a time, allowing examination of each study's influence on the overall effect size. Publication bias was assessed using funnel plots and Egger's regression test. A p‐value of <0.05 was regarded as statistically significant. Meta‐regression was not statistically powered due to < 10 studies per covariate.

### 2.7 Certainty Assessment

The quality of evidence was evaluated using the Grading of Recommendations Assessment, Development, and Evaluation (GRADE) approach [32], which examines five key domains: risk of bias, inconsistency, indirectness, imprecision, and publication bias. Based on this assessment, the overall certainty of the evidence is categorized into one of four levels: high, moderate, low, or very low.

## 3 Results

### 3.1 Study Selection

**Figure 1:**
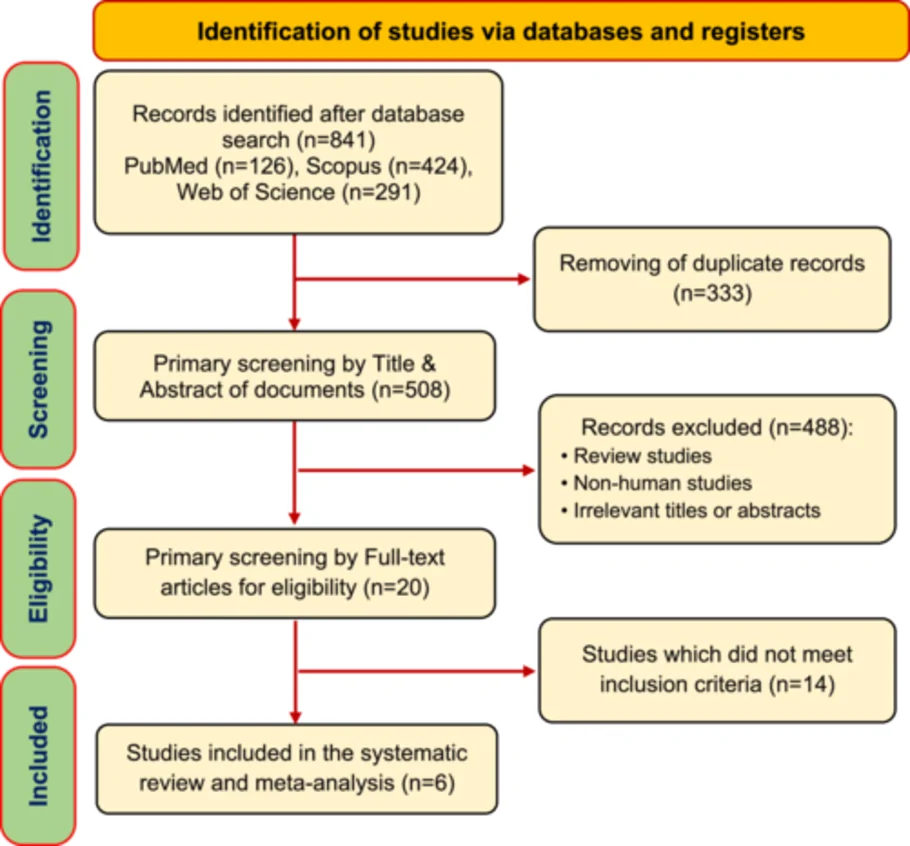
PRISMA Flow chart of the study selection process for inclusion studies in the systematic review.

In the initial search, a total of 841 publications were identified from PubMed (*n* = 126), ISI Web of Science (*n* = 291), and Scopus (*n* = 424). Of these, 333 articles were identified as duplicates, leaving 508 unique records for title and abstract screening. After an initial assessment against the inclusion criteria, 488 unrelated studies were excluded. Subsequently, 20 studies were reviewed in full text, and 16 were excluded due to insufficient data. As a result, six RCTs [33, 34, 35, 36, 37, 38] met the eligibility criteria for the current systematic review and meta‐analysis. The study selection process for inclusion in the systematic review is depicted in Figure 1.

### 3.2 Study Characteristics

In this meta‐analysis, six RCTs providing a total of 10 effect sizes were included to assess the effects of kiwifruit consumption on BP. Several trials yielded multiple effect sizes because they included more than one intervention arm. The studies included in the analysis were conducted in various countries, including Norway (n = 5) [33, 35], New Zealand (*n* = 3) [34, 37, 38], and Italy (*n* = 1) [36]. All studies employed a parallel design, except one study used a crossover design. Six of the studies were double‐blind, and three were single‐blind. The studies were published between 2012 and 2022. The duration of the follow‐up periods ranged from 7 to 20 weeks in the studies. The sample sizes included in the research varied from 20 to 106 participants. Five studies included only male participants [33, 34], while the remaining articles included both sexes. The interventions administered in the studies involved consuming either kiwifruits or green kiwifruits daily, in conjunction with a healthy diet. Four studies were conducted on smokers [33], two studies on subjects with periodontitis [36], one study on hypercholesterolemic men [34], one study on subjects with stage 1 hypertension [35], one study on healthy individuals [37], and one study on individuals with prediabetes [38]. The key attributes of the included studies are outlined in Table 1.

**Table 1:** Characteristics of included studies in the systematic review and meta‐analysis.

Study	Country	Study design	Participant	Sex	Sample size		Trial duration	Means age		Means BMI		Intervention		
					IG	CG		IG	CG	IG	CG	Type	Dose	CG
Karlsen et al. (2012) (A) [33]	Norway	RCT, Parallel, PC, DB	Smokers with optimal BP	M	12	11	8 weeks	57 ± 7	56 ± 6.75	24.7 ± 3.35	24.8 ± 2.2	Kiwifruits	3	Habitual diet
Karlsen et al. (2012) (B) [33]	Norway	RCT, Parallel, PC, DB	Smokers with normal‐high BP	M	12	12	8 weeks	57 ± 7	56 ± 6.75	24.7 ± 3.35	24.8 ± 2.2	Kiwifruits	3	Habitual diet
Karlsen et al. 2012 (C) [33]	Norway	RCT, Parallel, PC, DB	Smoker	M	33	34	8 weeks	57 ± 7	56 ± 6.75	24.7 ± 3.35	24.8 ± 2.2	Kiwifruits	3	Habitual diet
Karlsen et al. 2012 (D) [33]	Norway	RCT, Parallel, PC, DB	Smokers with Hypertension	M	9	11	8 weeks	57 ± 7	56 ± 6.75	24.7 ± 3.35	24.8 ± 2.2	Kiwifruits	3	Habitual diet
Gammon et al. (2013) [34]	New Zealand	RCT, Crossover, DB	Hypercholesterolemia	M	44	43	8 weeks	48 ± 6.7	48 ± 6.6	27.4 ± 2.87	27.3 ± 2.67	Kiwifruits + Healthy diet	2	Healthy diet
Svendsen et al. (2015) [35]	Norway	RCT, Parallel	Hypertension (stage 1)	M, F	51	55	8 weeks	55 ± 9	55 ± 9	26 ± 3.2	26 ± 3.2	Kiwifruits	3	Apple Royal Gala
Graziani et al. (2018) (A) [36]	Italy	RCT, Parallel, SB	Periodontitis	M, F	25	25	20 weeks	52.4 ± 9.2	50.4 ± 12.7	23.9 ± 4.4	24.4 ± 3.6	Kiwifruits	2	Control
Graziani et al. (2018) (B) [36]	Italy	RCT, Parallel, SB	Periodontitis	M, F	25	25	8 weeks	52.4 ± 9.2	50.4 ± 12.7	23.9 ± 4.4	24.4 ± 3.6	Kiwifruits	2	Control
Monro et al. (2022) [37]	New Zealand	RCT, Parallel, PC, DB	Healthy	M, F	20	22	7 weeks	21.9 ± 3.5	21.9 ± 2	22.4 ± 2.5	21.6 ± 3.4	Kiwifruits	2	Carbonated water
Mishra et al. (2022) [38]	New Zealand	RCT, Parallel, PC, SB	Prediabetes	M, F	17	15	12 weeks	55.3 ± 8.3	57 ± 10.9	30.5 ± 7.2	30.6 ± 5.7	Kiwifruits	2	Water

Abbreviations: BP, blood pressure; CG, control group; CO, controlled; DB, double‐blinded; F, female; IG, intervention group; M, male; NR, not reported; PC, placebo‐controlled; R, randomized; SB, single‐blinded.

### 3.3 Meta‐Analysis: Effect of Kiwifruit on SBP

**Figure 2:**
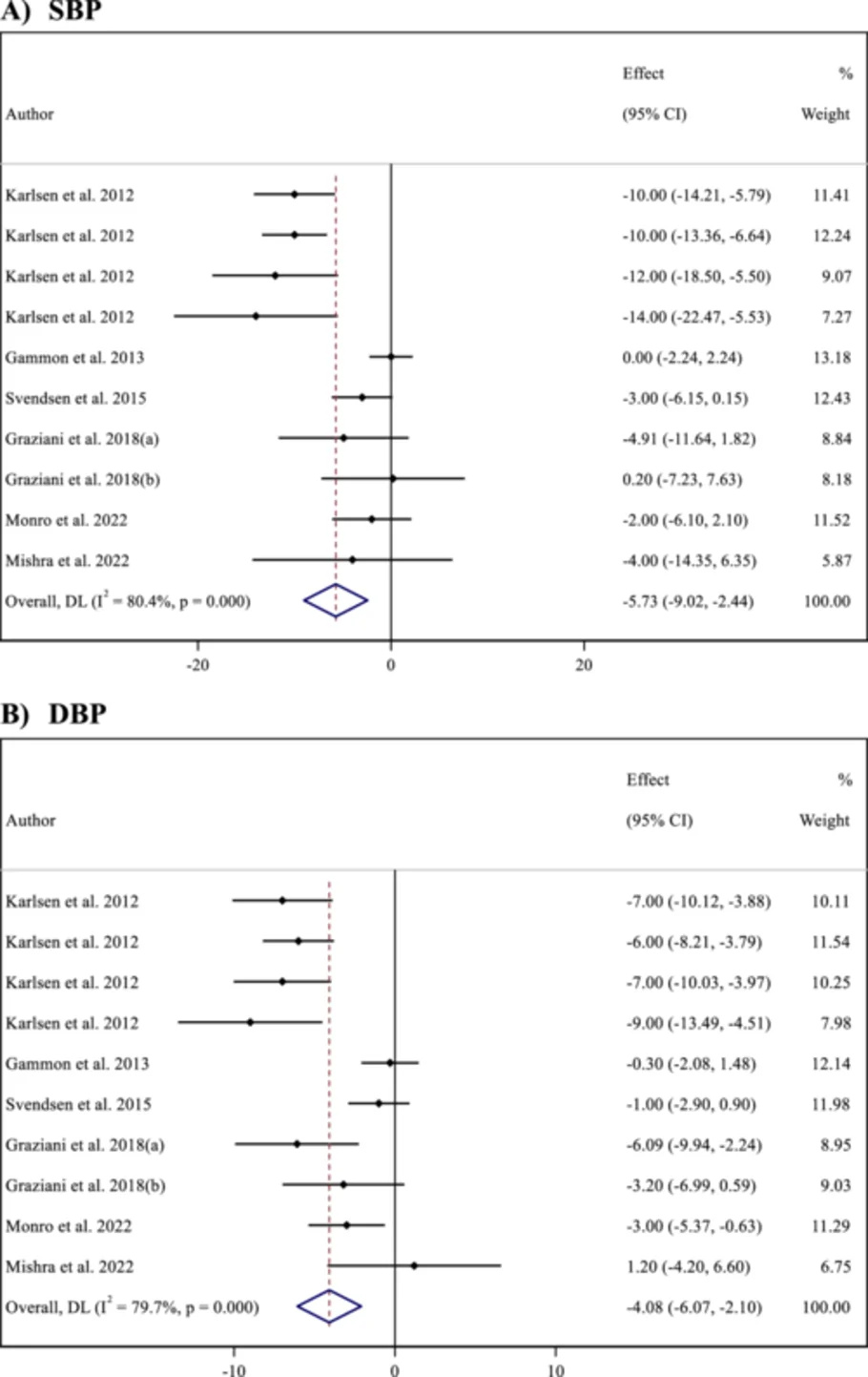
Forest plot detailing weighted mean difference (WMD) and 95% confidence intervals (CIs) for the effect of kiwi fruit intake on (A) systolic blood pressure (SBP) (mmHg) and (B) diastolic blood pressure (DBP) (mmHg) in adults.

In total, 10 effect sizes were analyzed to assess the impact of consuming kiwifruit on SBP. The combined effect size calculated using the random‐effects model demonstrated a significant decrease in SBP linked to kiwifruit consumption (WMD: −5.73 mmHg; 95% CI: [−9.02 to −2.44], *p* < 0.001). The research demonstrated marked heterogeneity across studies (*I* ^2^ = 80.4%, *p* < 0.001) (Figure 2A).

Subgroup analysis revealed that kiwifruit consumption significantly reduced SBP, more pronounced in participants with elevated BP (baseline SBP > 120 mmHg) (WMD: −5.67 mmHg; *p* = 0.004), while no significant effect was observed in those with SBP < 120 mmHg (*p* = 0.135), although the difference between subgroups was not statistically significant (*p* = 0.945). Regarding trial duration, studies lasting ≤ 8 weeks showed a significant reduction in SBP (WMD: −5.96 mmHg; *p* = 0.002), while those > 8 weeks did not (*p* = 0.107), with no significant difference between subgroups (*p* = 0.701). A significant subgroup effect was noted for dosage: 3 kiwifruits/day resulted in a substantial decrease in SBP (WMD: −9.10 mmHg; *p* < 0.001), whereas two kiwifruits/day showed no significant impact (*p* = 0.358; *p* for subgroup difference < 0.001). Additionally, individuals with normal BMI experienced a significantly greater reduction (WMD: −7.46 mmHg; *p* < 0.001) compared to overweight individuals (*p* = 0.263; *p* for subgroup difference = 0.005). The effect was also more prominent in males (WMD: −8.73 mmHg; *p* = 0.004) than in mixed‐sex groups (*p* = 0.016), with the difference approaching statistical significance (*p* = 0.063) (Table 2).

**Table 2:** Meta‐analysis findings for the effects of kiwifruit consumption on blood pressure in adults.

	No. of IR	WMD (95% CI)	*p* value	Heterogeneity		
				*p* heterogeneity	*I* ^2^	*p* between sub‐groups
Systolic blood pressure (SBP) (mmHg)						
Overall effect	10	−5.73 (−9.02, −2.44)	**0.001**	< 0.001	80.4%	
SBP						
< 120 mmHg	2	−5.98 (−13.82, 1.85)	0.135	0.008	86.0%	0.945
> 120 mmHg	8	−5.67 (−9.58, −1.77)	**0.004**	< 0.001	81.2%	
Trial duration (week)						
≤ 8 weeks	8	−5.96 (−9.71, −2.21)	**0.002**	< 0.001	84.8%	0.701
> 8 weeks	2	−4.64 (−4.64, 1.00)	0.107	0.885	0.0%	
Dose						
2	5	−0.84 (−2.64, 0.95)	0.358	0.611	0.0%	< 0.001
3	5	−9.10 (−13.10, −5.11)	**< 0.001**	0.004	73.8%	
Baseline BMI (kg/m^2^)						
Normal (18.5–24.9)	7	−7.46 (−11.07, −3.85)	**< 0.001**	0.004	68.7%	0.005
Overweight (25–29.9)	3	−1.31 (−3.62, 0.98)	0.263	0.269	23.9%	
Sex						
Both	5	−2.68 (−4.87, −0.50)	**0.016**	0.877	0.0%	0.063
Male	5	−8.73 (−14.73, −2.73)	**0.004**	< 0.001	90.5%	
Diastolic blood pressure (DBP) (mmHg)						
Overall effect	10	−4.08 (−6.07, −2.10)	**< 0.001**	< 0.001	79.7%	
DBP						
< 80 mmHg	3	−3.24 (−6.86, 0.36)	0.078	0.001	85.6%	0.573
> 80 mmHg	7	−4.51 (−7.02, −2.00)	**< 0.001**	< 0.001	77.0%	
Trial duration (week)						
≤ 8 weeks	8	−4.29 (−6.47, −2.11)	**< 0.001**	< 0.001	82.3%	0.675
> 8 weeks	2	−2.70 (−9.82, 4.42)	0.457	0.031	78.5%	
Dose						
2	5	−2.30 (−4.51, −0.09)	**0.041**	0.037	60.7%	0.070
3	5	−5.74 (−8.72, −2.75)	**< 0.001**	< 0.001	83.1%	
Baseline BMI (kg/m^2^)						
Normal (18.5–24.9)	7	−5.67 (−7.19, −4.15)	**< 0.001**	0.119	40.7%	< 0.001
Overweight (25–29.9)	3	−0.52 (−1.79, 0.73)	0.414	0.707	0.0%	
Sex						
Both	5	−2.49 (−4.42, −0.55)	**0.012**	0.104	47.9%	0.115
Male	5	−5.63 (−9.02, −2.23)	**0.001**	< 0.001	87.4%	

*Note:* Bold values indicate statistically significant (*p* < 0.05).

Abbreviations: BMI: body mass index; CI, confidence interval; IR, intervention arms; WMD, weighted mean differences.

### 3.4 Meta‐Analysis: Effect of Kiwifruit on DBP

Ten clinical trials were conducted to assess the impact of consuming kiwifruit on DBP. The combined effect size derived from a random‐effects model indicated a significant reduction in DBP due to kiwifruit consumption (WMD: −4.08 mmHg; 95% CI: [−6.07 to −2.10], *p* < 0.001). The studies exhibited a significant degree of heterogeneity (I^2^ = 79.7%, *p* < 0.001) (Figure 2B).

In subgroup analysis, participants with baseline DBP > 80 mmHg experienced a significant reduction (WMD: −4.51 mmHg; *p* < 0.001), whereas those with DBP < 80 mmHg did not (*p* = 0.078), but the difference between these groups was not statistically significant (*p* = 0.573). Trials lasting ≤ 8 weeks showed significant improvements (WMD: −4.29 mmHg; *p* < 0.001), while longer trials did not (*p* = 0.457), and no significant subgroup difference was detected (*p* = 0.675). A dose of 3 kiwifruits/day produced a stronger effect (WMD: −5.74 mmHg; *p* < 0.001) compared to 2 kiwifruits/day (WMD: −2.30 mmHg; *p* = 0.041), though the subgroup difference was not statistically significant (*p* = 0.070). Normal‐weight participants (BMI: 18.5–24.9) saw a significantly greater reduction (WMD: −5.67 mmHg; *p* < 0.001) than overweight individuals (*p* = 0.414; p for subgroup difference < 0.001). Similarly, the reduction was more significant in males (WMD: −5.63 mmHg; *p* = 0.001) than in mixed‐sex groups (*p* = 0.012), although the subgroup difference was not statistically significant (*p* = 0.115) (Table 2).

### 3.5 Publication Bias and Sensitivity Analyses

**Figure 3:**
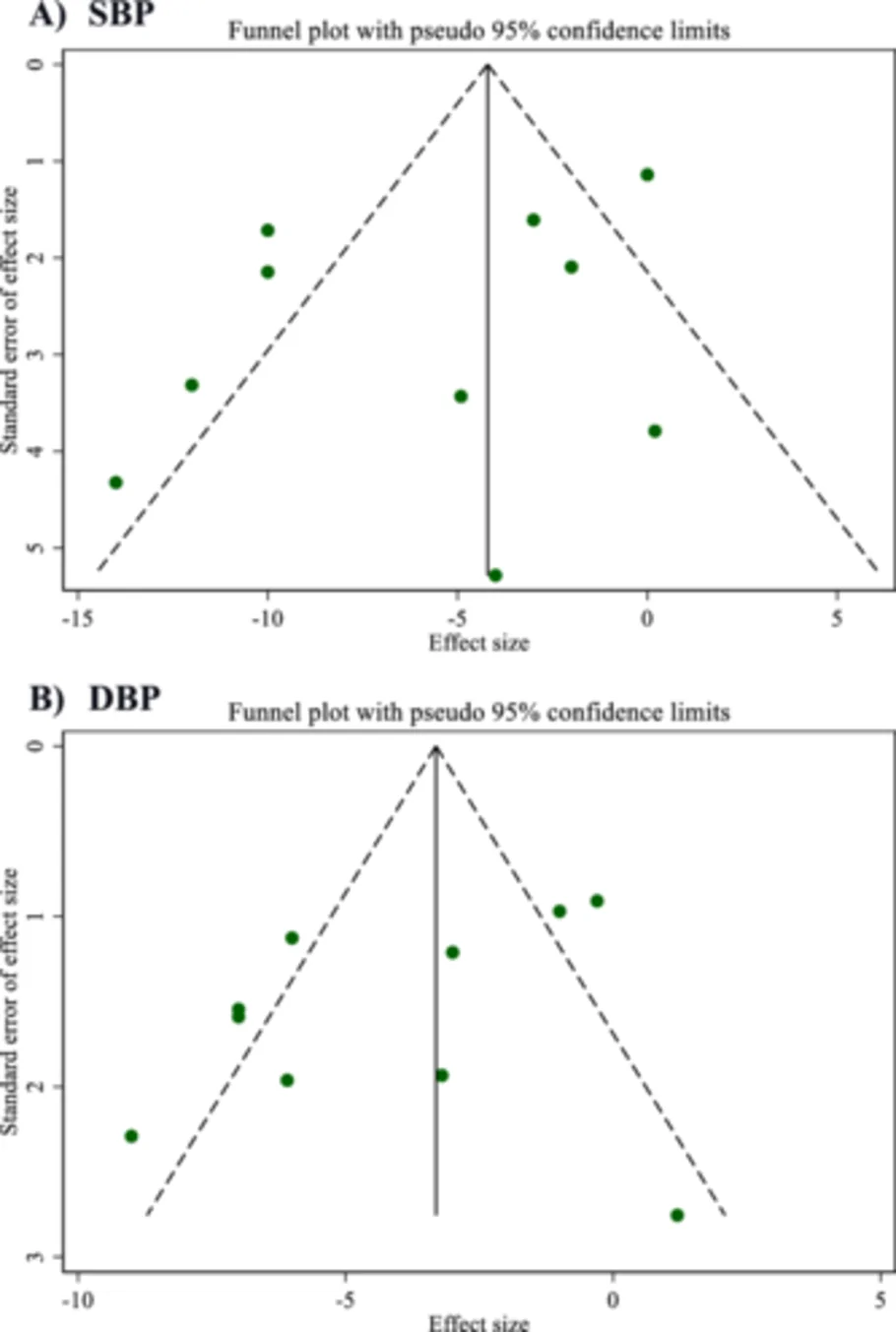
Funnel plots of publication bias for the effect of kiwi fruit intake on (A) systolic blood pressure (SBP) (mmHg) and (B) diastolic blood pressure (DBP) (mmHg) in adults.

Evaluation of funnel plots, along with Egger's regression test, revealed no evidence of publication bias for either SBP (*p* = 0.188) or DBP (*p* = 0.154) (Figure 3). Sensitivity analyses, conducted by sequentially removing individual studies, demonstrated that no single study significantly altered the overall effect sizes for SBP or DBP, indicating the robustness and stability of the pooled results.

### 3.6 Risk of Bias Assessment

The risk‐of‐bias assessment for the included studies is summarized in Table 3. Out of the six RCTs evaluated, five were classified as having an overall low risk of bias, including studies by Karlsen et al. (2012), Gammon et al. (2013), Svendsen et al. (2015), Monro et al. (2022), and Mishra et al. (2022). These studies demonstrated generally low or unclear risk across most domains, with no more than two domains rated as high risk. In contrast, the study by Graziani et al. (2018) was assessed as having a high overall risk of bias due to high‐risk ratings in more than two domains, particularly in areas related to blinding and other sources of bias. This suggests that, overall, the quality of evidence from the included studies is reasonably reliable, with only one study contributing potential methodological concerns.

**Table 3:** Risk of bias assessment.

Study	D1	D2	D3	D4	D5	D6	D7	Overall
Karlsen et al. (2012) [33]	U	L	L	H	L	U	L	Low risk
Gammon et al. (2013) [34]	L	L	L	L	L	U	L	Low risk
Svendsen et al. (2015) [35]	U	L	L	H	U	U	L	Low risk
Graziani et al. (2018) [36]	L	L	H	H	H	U	L	High risk
Monro et al. (2022) [37]	L	L	L	L	L	U	L	Low risk
Mishra et al. (2022) [38]	L	L	L	L	H	U	L	Low risk

*Note:* D1: Random sequence generation, D2: Allocation concealment, D3: Selective reporting, D4: Other sources of bias, D5: Blinding (participants and personnel), D6: Blinding (outcome assessment), D7: Incomplete outcome data, L: low risk of bias; H: high risk of bias; U: unclear risk of bias. General Low risk < 2 high risk. General moderate risk = high risk. General high risk > 2 high risk.

### 3.7 GRADE Assessment

The GRADE evaluation rated the quality of evidence for both SBP and DBP outcomes as moderate. Although no serious concerns were noted regarding risk of bias, indirectness, imprecision, or publication bias, the evidence was downgraded due to very serious inconsistency, primarily attributed to high heterogeneity across studies (*I* ^2^ > 75%). This variability suggests that the treatment effect may differ across populations or study conditions (Table 4).

**Table 4:** GRADE profile for the effects of kiwifruit consumption on blood pressure in adults.

Outcomes	Risk of bias	Inconsistency	Indirectness	Imprecision	Publication bias	Quality of evidence
***SBP***	No serious limitation	Very serious limitation^1^	No serious limitation	No serious limitation	No serious limitation	⊕⊕⊖⊖ Moderate
***DBP***	No serious limitation	Very serious limitation^1^	No serious limitation	No serious limitation	No serious limitation	⊕⊕⊖⊖ Moderate

*Note:* There is high heterogeneity (*I* ^2^ > 75%).

Abbreviations: DBP, diastolic blood pressure; SBP, systolic blood pressure.

## 4 Discussion

To our knowledge, this is the first systematic review and meta‐analysis to evaluate the effects of kiwifruit consumption on BP using evidence derived exclusively from RCTs. Our findings indicate that kiwifruit consumption significantly reduced both SBP and DBP compared with the control group. These results suggest that kiwifruit may contribute to BP management. However, the evidence is based on only six RCTs. Therefore, the precision and generalizability of the pooled estimates remain limited, and the findings should be interpreted with caution until they are confirmed by larger, well‐designed, adequately powered multicenter RCTs.

The substantial heterogeneity observed for both SBP and DBP outcomes may be attributable to differences in study populations, including smokers, individuals with hypertension, hypercholesterolemia, periodontitis, and prediabetes, as well as variations in kiwifruit dosage, intervention duration, baseline BP levels, and participant BMI. Subgroup analyses indicated that dose and BMI explained part of the between‐study variability. Although sensitivity analyses confirmed the stability of the pooled estimates, the high heterogeneity reduces confidence in the magnitude of the observed effects and suggests that the findings should be interpreted cautiously.

Hypertension remains one of the most modifiable contributors to early‐onset morbidity and mortality. Achieving optimal BP control is particularly advantageous for individuals with hypertension or pre‐existing cardiovascular conditions, given their elevated risk profiles [39]. Moreover, the consumption of a diverse range of fruits, vegetables, and herbs rich in essential macro‐ and micronutrients has been linked to favorable effects on cardiovascular risk markers across various population groups [40, 41, 42].

Prior studies have indicated that kiwifruit consumption can reduce CVD risk factors, including platelet aggregation, high BP, and lipid profiles, in both healthy individuals and smokers [33, 43]. A 2021 meta‐analysis found that adhering to plant‐based dietary patterns with minimal animal product consumption may reduce both SBP and DBP [44]. Furthermore, numerous studies have demonstrated that increased consumption of fruits and vegetables is associated with lower BP and a reduced incidence of CVD, including coronary heart disease (CHD). However, it is also hypothesized that specific varieties of fruits or vegetables may exert more pronounced effects on BP, potentially due to unique bioactive constituents or other nutritional properties [45, 46].

Kiwifruit is a nutrient‐dense fruit rich in phytonutrients, antioxidants, and enzymes that play important roles in human metabolism. Its antioxidant compounds, including polyphenols, ascorbic acid (vitamin C), carotenoids, and tocopherols, help neutralize oxidative stress by reducing hydrogen peroxide, binding metal ions, and deactivating reactive oxygen species such as superoxide and singlet oxygen [47]. These mechanisms collectively protect the body from harmful free radicals. In addition to its antioxidant content, kiwifruit provides substantial dietary fiber, with a single fruit offering about 10% of the recommended daily intake. High fiber intake is linked to improved lipid profiles and a reduced risk of cardiovascular diseases, including coronary heart disease and myocardial infarction [15, 47].

Kiwifruit is also notable for its high potassium content, an essential mineral known for its BP‐lowering properties. Epidemiological studies have consistently shown an inverse association between potassium intake and the prevalence of hypertension [48]. Kiwifruit contributes favorably to the sodium‐to‐potassium (Na^+^/K^+^) ratio, which is critical for maintaining cardiovascular health [49]. Diets like the DASH (Dietary Approaches to Stop Hypertension) diet, which emphasize high fiber and potassium with low salt and fat intake, have been shown to significantly lower arterial BP [50]. The antihypertensive effect of potassium is attributed to several physiological mechanisms, including vasodilation, increased glomerular filtration rate, reduced renin secretion, decreased renal sodium reabsorption, and inhibition of platelet aggregation [44].

Kiwifruit is also a rich source of vitamin C, a nutrient that has been extensively studied for its vascular protective properties [15]. Numerous clinical investigations have consistently demonstrated an inverse relationship between vitamin C intake, systemic ascorbic acid concentrations, and the risk of developing hypertension [51]. Additionally, studies have shown that kiwifruit consumption can decrease angiotensin‐converting enzyme (ACE) activity, suggesting that specific bioactive components in kiwifruit may inhibit this enzyme. Given that ACE plays a central role in BP regulation by producing the vasoconstrictor angiotensin II within the renin–angiotensin system, modulating its activity is a critical therapeutic target in hypertension management [33, 52].

Current evidence from previous reviews suggests that incorporating kiwifruit into the diet may offer benefits for individuals with hypertension, largely due to its potential antihypertensive effects [33, 53, 54, 55]. For example, Svendsen et al. [35] conducted a study involving participants with either normal BP or stage 1 hypertension, who consumed three kiwifruits daily (intervention) or one apple daily (control) over 8 weeks. The results indicated that kiwifruit intake was associated with significant reductions in SBP (−3.6 mmHg) and DBP (−1.9 mmHg) compared with the apple group. Conversely, Gammon et al. [56] investigated men with hypercholesterolemia, comparing those who consumed two kiwifruits daily alongside a healthy diet to those who followed only a healthy diet. This study did not find any improvements in BP, which may be partly explained by the fact that only two participants had hypertension.

Additionally, a previous systematic review and meta‐analysis by Suksomboon et al. [57] evaluated the effects of kiwifruit on multiple cardiometabolic outcomes in individuals with cardiovascular risk factors and reported no significant effects on BP. In contrast, the present review focused specifically on BP outcomes, incorporated additional RCTs published after the previous review, and conducted subgroup analyses to explore potential effect modifiers. These methodological differences may explain the discrepancy between the findings.

Kiwifruit may be a practical, natural dietary option for lowering BP, particularly for individuals not yet on medication. Its favorable safety profile and cardiovascular benefits suggest its potential as a complementary dietary strategy in the prevention and management of hypertension.

This meta‐analysis is the first to comprehensively evaluate the effect of kiwifruit consumption on BP using RCT data, thereby strengthening the evidence base through rigorous methodology, subgroup analyses, and GRADE assessment. The findings indicate clinically relevant reductions in both SBP and DBP, particularly among individuals with elevated BP, normal BMI, and those consuming higher kiwifruit doses.

Despite these strengths, several limitations should be acknowledged. The relatively small number of available RCTs may have reduced statistical power, limited the assessment of publication bias, and constrained the generalizability of the findings. In addition, most trials were short‐duration, and considerable variability in study design, participant characteristics, and intervention protocols, along with substantial between‐study heterogeneity in several outcomes, may have influenced the pooled estimates and reduced confidence in the observed effect sizes. Furthermore, the external validity of the results may be limited, as many included studies were conducted in specific populations, including male smokers and individuals with hypercholesterolemia, periodontitis, prediabetes, or elevated BP; therefore, the findings may not be fully generalizable to the broader adult population or to women and ethnically diverse groups.

Although subgroup analyses suggested that factors such as dose, BMI, sex, and baseline BP may modify the observed effects, these results should be interpreted with caution, as several subgroup categories included few studies and participants, increasing the risk of chance findings and reducing statistical power. Future research should therefore focus on large‐scale, long‐term, well‐designed RCTs in more diverse populations to confirm these findings and further elucidate the underlying biological mechanisms of the BP–lowering effects of kiwifruit consumption.

## 5 Conclusion

In conclusion, the available evidence suggests that kiwifruit consumption may represent a safe, natural, and potentially effective dietary strategy for lowering BP, particularly among individuals with elevated baseline BP and a normal BMI, especially when consumed at higher doses (e.g., three kiwifruits per day). The observed reductions in both SBP and DBP support a potential antihypertensive effect of kiwifruit. However, the certainty of the evidence remains limited due to the small number of available RCTs, substantial between‐study heterogeneity, variation in participant characteristics, and relatively short intervention durations. Larger, well‐designed RCTs with longer follow‐up periods are needed to confirm these findings and establish more definitive clinical recommendations.

## Author Contributions

**Shirin Ghotboddin Mohammadi:** writing – original draft, investigation, conceptualization, funding acquisition, visualization, validation, methodology, data curation, software, resources. **Neda Haghighat:** methodology, validation, visualization, writing – review and editing, funding acquisition, writing – original draft, investigation, conceptualization, data curation, software, resources. **Azadeh Dehghani:** methodology, visualization, validation, writing – original draft, funding acquisition, conceptualization, investigation, data curation, software, resources. **Mohammad Mahdi Rafiei:** data curation, software. **Pedram Pam:** writing – original draft, funding acquisition, investigation, conceptualization, validation, methodology, writing – review and editing, visualization, resources, software. **Mohammadreza Amirkhan‐Dehkordi:** visualization, validation, methodology, writing – original draft, funding acquisition, investigation, conceptualization, software. **Mohammad Ali Goudarzi:** software, writing – original draft, investigation, funding acquisition, conceptualization, methodology, validation, visualization, resources. **Omid Asbaghi:** data curation, formal analysis, supervision, resources, project administration, validation, methodology, conceptualization, investigation, visualization, funding acquisition, software, writing – original draft. **Moslem Naderian:** writing – review and editing, writing – original draft, funding acquisition, investigation, conceptualization. **Ali Hosseini:** writing – review and editing, writing – original draft, investigation, funding acquisition. **Mehdi Karimi:** writing – original draft, funding acquisition, investigation, methodology, validation, writing – review and editing, visualization, software, supervision, resources, project administration, conceptualization.

## Funding

The authors have nothing to report.

## Ethics Statement

The authors have nothing to report.

## Conflicts of Interest

The authors declare no conflicts of interest.

## Transparency Statement

The Corresponding author (*Mehdi Karimi*) affirms that this manuscript is an honest, accurate, and transparent account of the study being reported; that no important aspects of the study have been omitted; and that any discrepancies from the study as planned have been explained.

## Data Availability

All data used in this meta‐analysis were extracted from published studies. The datasets supporting the findings of this study are available from the sources cited in the manuscript. Additional information can be provided by the corresponding author upon reasonable request.
